# RETRACTED: Ji, W.; Chen, X. TRUST: A Novel Framework for Vehicle Trajectory Recovery from Urban-Scale Videos. *Sensors* 2022, *22*, 9948

**DOI:** 10.3390/s24113672

**Published:** 2024-06-06

**Authors:** Wentao Ji, Xing Chen

**Affiliations:** College of Electronics and Information Engineering, Sichuan University, Chengdu 610064, China

The *Sensors* Editorial Office retracts the article, “TRUST: A Novel Framework for Vehicle Trajectory Recovery from Urban-Scale Videos” [1], cited above.

Following publication, concerns were brought to the attention of the publisher regarding the overlap between this article [1] and an earlier thesis published in another language [2], authored by an unconnected academic.

Adhering to our complaints procedure, an investigation was conducted by the Editorial Office and the Editorial Board, and this confirmed a significant overlap of text, images and content. Given the extent of the overlap, the Editorial Office and Editorial Board have decided to retract this article [1], as per MDPI’s retraction policy (https://www.mdpi.com/ethics#_bookmark30 (accessed on 3 April 2024)) and in line with the Committee on Publication Ethics retraction guidelines (https://publicationethics.org/retraction-guidelines (accessed on 3 April 2024)).

This retraction was approved by the Editor-in-Chief of *Sensors*.

The authors did not agree with this retraction.
